# Considerations for Optimization of High-Throughput Sequencing Bioinformatics Pipelines for Virus Detection

**DOI:** 10.3390/v10100528

**Published:** 2018-09-27

**Authors:** Christophe Lambert, Cassandra Braxton, Robert L. Charlebois, Avisek Deyati, Paul Duncan, Fabio La Neve, Heather D. Malicki, Sebastien Ribrioux, Daniel K. Rozelle, Brandye Michaels, Wenping Sun, Zhihui Yang, Arifa S. Khan

**Affiliations:** 1 GSK, 1330 Rixensart, Belgium; avis.quest@gmail.com; 2 Biogen Inc., Research Triangle Park, NC 27709, USA; cassandra.braxton@biogen.com; 3 Analytical Research and Development, Sanofi Pasteur, Toronto, ON M2R 3T4, Canada; Robert.Charlebois@sanofi.com; 4 Merck & Co. Inc., West Point, PA 19486, USA; paul_duncan@merck.com; 5 Merck KGaA, 10010 Torino, Italy; fabioinusa@gmail.com; 6 WuXi AppTec, Philadelphia, PA 19112, USA; Heather.Malicki@wuxiapptec.com (H.D.M.); kathryn.sun@gmail.com (W.S.); 7 Genedata AG, 4053 Basel, Switzerland; sebastien.ribrioux@genedata.com; 8 Radiant Systems, Inc., Plainfield, NJ 07080, USA; dan.rozelle@gmail.com; 9 Analytical Research and Development: Microbiology, Pfizer Inc., Andover, MA 01810, USA; Brandye.Michaels@pfizer.com; 10 Office of Applied Research and Safety Assessment, Center for Food Safety and Applied Nutrition, U.S. Food and Drug Administration, Laurel, MD 20708, USA; Zhihui.Yang@fda.hhs.gov; 11 Office of Vaccines Research and Review, Center for Biologics Evaluation and Research, U.S. Food and Drug Administration, Silver Spring, MD 20993, USA; Arifa.Khan@fda.hhs.gov

**Keywords:** high-throughput sequencing, bioinformatics pipeline, adventitious virus detection

## Abstract

High-throughput sequencing (HTS) has demonstrated capabilities for broad virus detection based upon discovery of known and novel viruses in a variety of samples, including clinical, environmental, and biological. An important goal for HTS applications in biologics is to establish parameter settings that can afford adequate sensitivity at an acceptable computational cost (computation time, computer memory, storage, expense or/and efficiency), at critical steps in the bioinformatics pipeline, including initial data quality assessment, trimming/cleaning, and assembly (to reduce data volume and increase likelihood of appropriate sequence identification). Additionally, the quality and reliability of the results depend on the availability of a complete and curated viral database for obtaining accurate results; selection of sequence alignment programs and their configuration, that retains specificity for broad virus detection with reduced false-positive signals; removal of host sequences without loss of endogenous viral sequences of interest; and use of a meaningful reporting format, which can retain critical information of the analysis for presentation of readily interpretable data and actionable results. Furthermore, after alignment, both automated and manual evaluation may be needed to verify the results and help assign a potential risk level to residual, unmapped reads. We hope that the collective considerations discussed in this paper aid toward optimization of data analysis pipelines for virus detection by HTS.

## 1. Introduction

Eukaryotic cells have been used to produce biological medicinal products since the early 1950s, based on their broad susceptibility to grow vaccine viruses. However, their susceptibility for infection by a wide range of viruses can also support growth of unwanted adventitious viruses. Therefore, safeguards against infection by extraneous viruses are implemented during product manufacture, including viral clearance steps, when possible; extensive in-process testing using analytical methods; and also, when using qualified raw materials [1,2]. Analytical methods for general virus detection include animal- and cell culture-based assays, as well as specific assays for detection of virus families, such as reverse transcriptase (RT) assays to detect retroviruses, and targeted or degenerate polymerase chain reaction (PCR) assays to detect specific viruses [1,2]. High-throughput sequencing (HTS) (also referred to as massively parallel sequencing (MPS) or next generation sequencing (NGS)) has recently emerged as a new technology that can enhance virus detection, due to its ability to detect known and novel viruses [3,4]. The capabilities of HTS for broad virus detection in biologics are evidenced by the unexpected detection of viruses in a vaccine and in production bioreactors, and the discovery of novel viruses in various cell lines, including insect and mammalian [5,6,7,8]. Furthermore, the use of HTS for adventitious virus detection to supplement or, possibly, in the future to replace, some of the current virus testing methods were discussed at the “NGS for Adventitious Virus Detection in Biologics” conference held in Rockville Maryland, U.S.A. by the International Alliance for Biological Standardization (IABS) and the U.S. Food and Drug Administration (FDA) on 26–27 October 2017 (meeting report in preparation).

HTS is recognized as a powerful tool for virus discovery in biological sciences and related industries. Additionally, although the technology continues to evolve, use of HTS is increasing due to the availability of established platforms with higher throughput and reduction in cost. However, due to the technical and bioinformatics complexities of the method, there is a need for standardization of the multiple steps for evaluation of biologics. Variability in HTS results can arise from any of these critical components of the system, including sample preparation involving the sample matrix and nucleic acid sample type (DNA, RNA, or both); nucleic acid amplification method; library preparation; the sequencing platform; and decisions related to the bioinformatics analysis, such as whether to assemble the HTS reads, and whether to extend the analysis to the protein sequence level to include detection of more divergent, still genetically uncharacterized, “novel” viral genomes. Moreover, as with any nucleic acid-based virus detection method, HTS only indicates the presence of viral sequences, and needs further follow-up to experimentally confirm the results and exclude potential laboratory and reagent contamination, as well as to assess the biological relevance and significance of the result for decision-making. The Advanced Virus Detection Technologies Interest Group (AVDTIG) was formed to address some of these challenges and provide a public forum to discuss the experimental and computational issues relating to adventitious virus detection among scientists from industry, government, academia, technology providers, and others [9]. Additionally, there are metagenomics analysis tools that can be used for global nucleic acid evaluation, including viruses [10]. Clearly, there has been a rapid proliferation of these tools, reflecting significant progress in this space, but also presenting a real challenge in deciding what is necessary and sufficient for potentially regulated applications. Nonetheless, the published pipelines provide a good illustration of approaches and features that are important to consider, and many of these are discussed in this paper.

This paper presents our current thinking on bioinformatics pipelines with focus on the choices made in the analysis pipeline—including, but not limited to, sequences, aligners, databases, assembly, and how unmapped reads are managed. Discussions on sample selection and preparation, as well as virus spiking to determine sensitivity, are presented in the context of their influence on the pipeline analysis and details are discussed in another paper from AVDTIG members [11].

## 2. Factors Influencing Sensitivity of Virus Detection

HTS can provide a sensitive and rapid platform for broad virus detection and identification in various biological matrices, without prior sequence knowledge of potential viral contaminants, in contrast to other nucleic acid-based methods, such as quantitative PCR (qPCR), or hybridization-based targeted nucleic acid assays [12].

There are several factors that can influence the sensitivity of virus detection by HTS in a biological sample. Some important considerations are discussed as follows.

### 2.1. Upstream Preparation of the Biological Sample

HTS typically generates millions of reads with one run, and these reads may consist of host-derived cellular sequences. The amount of nucleic acid in a biological test sample can vary based upon processing steps prior to nucleic acid extraction. High background from cellular nucleic acids will reduce the sensitivity of HTS virus detection [13,14]. In most cases, the cellular nucleic acids are the most crucial factor for influencing the sensitivity of virus detection, since they typically dominate the viral read number [11,15,16]. Reducing the cellular nucleic acid, such as by nuclease treatment prior to sequencing, may enhance the sensitivity of virus detection in downstream bioinformatics analysis.

### 2.2. Sequencing

It has been generally accepted that generating more reads in a sequencing run increases the probability of detecting the presence of low-level virus. A study has shown that both the quantity and purity of viral RNA (relative to background or host RNA) can affect viral read numbers and sequencing coverage of viral genome [13]. In general, increasing the number of reads will increase both the average depth and breadth of coverage of the viral genomes in the sample, thus increasing the confidence level of the detection. However, the sensitivity of virus detection depends on the size of the virus genome, the type of virus, and the amount of background nucleic acids. Optimally, a single viral genome in the tested sample should be detectable, but this may be represented by a few reads and, therefore, would need follow-up for confirmation.

### 2.3. Bioinformatics Pipeline and Databases

Various sequence mapping algorithms, such as Bowtie2 [17], BWA [18], BLAST [19], BLAT [20], and DIAMOND [21], are freely available and, therefore, the most frequently applied bioinformatics analysis approaches. The parameters defined in the analysis pipelines, as well as the threshold stringency applied, are also important factors in terms of making a true-positive call. With regard to the viral database, the more up-to-date and comprehensive the viral sequence collection, the greater the likelihood to detect and identify the presence of virus. More details on this topic are discussed in later sections.

**Figure 1:**
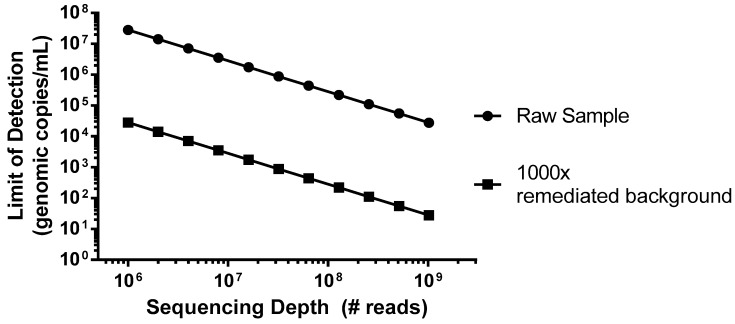
Predicted sensitivity for porcine circovirus (PCV) at a given sequencing depth, with and without incorporation of steps to reduce cellular nucleic acid background. Calculated deep sequencing results of a sample composed of 1 × 10^7^ CHO cells/mL (2.4 × 10^9^ bp genome) spiked with PCV type 2 (PCV-2) (1.7 × 10^3^ bp genome). For a given sequencing depth, the viral concentration at which a single viral read is expected to be obtained is shown. For example, if we would like to ensure sensitivity for PCV-2 above 1 × 10^6^ copies/mL, we would need to generate at least 1 × 10^7^ total reads. The effect of reducing background CHO cell DNA by 1000-fold is shown in the graph by closed squares. Under these conditions, we can expect a similar sensitivity with only 3 × 10^5^ total reads.

As an example of a worst case scenario for detection of virus contamination, we have simulated a spiking study using porcine circovirus (PCV), the smallest virus particle and genome size, with a single-strand circular DNA genome [22], which has potential for Chinese hamster ovary (CHO) cell infection [23]. This virus would be particularly difficult to detect due to its small genome size of only ~1.7 kb that encodes for just two proteins, and the amount of virus (copies) relative to the host DNA nucleotides. In this example, we can consider a typical harvest day CHO density of 1 × 10^7^ cells/mL, which would introduce 5 × 10^16^ bp of background DNA per mL. As shown in Figure 1 (closed circles), without incorporation of steps to reduce the background, even an entire Illumina HiSeq flow cell run, generating 2.5 × 10^9^ reads in a paired-end mode (for example 2 × 100 bp) would not likely produce a single PCV read below a sensitivity of 1 × 10^4^ genomes/mL. While mechanisms of target enrichment and background removal are beyond the scope of this paper [11], when we explore the effect of improving this signal-to-noise ratio (S/N) by 1000× (Figure 1, closed squares), similar levels of sensitivity could then be obtained using a more modest platform (MiSeq, ~1.2 × 10^7^ paired-end reads).

## 3. Sequencing Platform and Output Files

There are several sequencing platforms currently available for short reads, including instruments from Illumina (NovaSeq, HiSeq, NextSeq, MiSeq) and Life Technologies (Ion Torrent PGM and Proton). Additional platforms for long reads, such as those of Pacific Biosciences (Sequel System and PacBio RS II) have recently been established, and some are still evolving, such as Oxford Nanopore Technologies (MinION and PromethION). Each of the instruments have strengths and limitations, mostly related to cost, number of reads, read length, error rate, run duration, and complexity of library construction, but all have been shown to detect viruses in biological materials [3,24,25]. The selection of the sequencing platform is an important consideration for the bioinformatics analysis. Indeed, short read platforms can generate more accurate sequences but more difficult to assemble in some cases, and long read platforms currently have a higher rate of sequence error, but more accurate assembled sequences in the single read. Therefore, a hybrid analysis approach can be used to include the short reads for correction of base error in the long reads [26].

The number of sequencing reads will impact the sensitivity of the assay. As such, the sequencers yielding the highest number of reads per sample, whether single- or paired-end, will have the best chance of detecting a low-level contaminant. Therefore, for biological samples, such as most manufacturers’ master and working cell banks, a sequencing platform that will yield the highest level of coverage should be used to increase the ability to detect a potential low-level virus contaminant. In the event that a biological material is suspected to be contaminated with a high level of adventitious virus, the sequencing platform may be less important as the viral nucleic acid is expected to be more easily detectable. However, more reads will increase the depth and the coverage of the sequencing, and the likelihood of generating the full viral genome.

Data obtained from different sequencing platforms may be analyzed using a variety of bioinformatics tools and programs. The more frequently used sequence file formats today are FASTQ and FASTA. FASTQ, which includes information about the base calling quality, is usually used by programs such as Bowtie2 [17] or BWA [18], to map/align unassembled reads against the reference sequences, while FASTA, which consists of plain sequence information, is more suitable for aligning assembled reads using algorithms such as BLAST [19] or BLAT [20]. However, some sequencing technologies have their own format, e.g., Oxford Nanopore and Pacific Bioscience are in HDF5 file format: FAST5 in case of Nanopore, and bas.h5/bax.h5 and cmp.h5 for Pacific Bioscience. These are later converted to FASTA and FASTQ formats for downstream analysis.

FASTQ is a popular file format since it incorporates the nucleotide sequences and the Phred quality score of each base call in the file. The Phred quality score relates to the base-calling error probability, which is used to measure the quality of the identification of each nucleotide generated by automated DNA sequencing. In this case, the base call quality and alignment/mapping quality scores obtained during sequence alignment can both be used as filtering thresholds. Typically, a Phred score of 20 is used as a threshold, with probability of error of 0.01.

## 4. Data Analysis Pipeline Design

Virus detection using HTS datasets of biological samples is essentially a viral metagenomic analysis. Currently, several commercial laboratories are developing viral metagenomic analyses as a service for the biopharma industry. In addition, custom pipelines are being developed within some biopharma companies. Also, several non-commercial laboratories have developed metagenomic analysis pipelines that are at least nominally targeted at clinical diagnostics laboratories or for general environmental applications, such as Taxonomer [27], SURPI [28], Metavir [29], MEGAN [30], Kraken [31], Virome [32], CensuScope [33], ViromeScan [34], Kaiju [35], or for biodefense, such as LMAT [36]. Commercial software developers have also contributed to this space by developing various tools to facilitate bioinformatics analysis of large datasets and using different HTS platforms, such as OMICTools [37], CLCBio [38], Genedata Selector [39], Archetype Software [40], and CosmosID [41].

**Figure 2:**
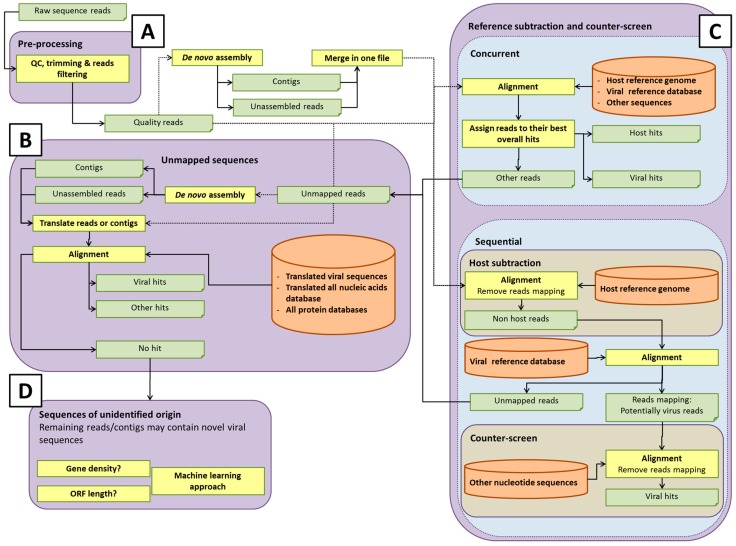
Potential pipelines for HTS data analysis for virus detection. Any given pipeline might use one or a combination of such paths, or others. See text for details. (**A**) Pre-processing, (**B**) Unmapped sequences, (**C**) Reference subtraction and counter-screen, and (**D**) Sequences of unidentified origin.

Some potential pathways for bioinformatics analysis pipelines are illustrated in Figure 2. The details of each process are discussed in the sections below. Within each element of the analysis pipeline, specific choices can affect the detection of rare or novel viral sequences, and the computational efforts involved [42]. For instance, the total number of short reads or assembled contigs, and the size of databases used for host sequence removal (if used) or data analysis, will directly influence the computational time and computer resources used. Analysis of all-frame-translated protein sequences may afford a greater chance of detecting novel viral sequences that are not represented in the nucleotide database. Developers of pipelines must make choices that afford adequate sensitivity for detection of viruses at acceptable computational cost. It is prudent to save any unmapped reads for future analysis as databases are updated with new virus sequences.

In the rest of this paper, we discuss some of the specific elements of the bioinformatics analysis pipelines, and offer our current thinking on their strengths and limitations.

### 4.1. Sequence Read Pre-Processing, Assembly, and Alignment

Sequencing reads may suffer from quality issues, such as low-scoring base calls or the presence of platform-specific adapter sequences. A variety of tools are available to evaluate the existence of such issues, such as FastQC [43] or MEEPTOOLS [44], which generates a number of useful graphical plots, for example, displaying quality score distribution against read base position. There are additional tools that can be further employed to simultaneously remove these adapter sequences and trim away bases with low Phred scores, such as Trimmomatic [45], ngsShoRT [46], CLC Genomic Workbench, or Illumina CASAVA.

After quality control, short reads are usually aligned to a reference sequence or to a database of reference sequences. Another possible strategy for virus identification is de novo assembly of the reads without any prior alignment to a database (Figure 2A). Based on experimental design, the sequence read can be either single- or paired-end. Paired-end reads offer more accurate sequences, however, if paired-end sequencing is performed, one should keep the read pair information in the mapping/alignment/assembly process.

There are several algorithms that can be employed by the aligners, however one of the most used by the aligners is the Burrows–Wheeler transform, that helps to reduce the memory effort for short-sequence alignment. Generally, the alignment consists in a pre-processing of the reference sequence to generate an index and the subsequent mapping of the reads against the indexed reference sequences. Currently the most widely used aligners are Bowtie2 [17], BWA [18], and SOAP2 [47]. These aligners usually accept only limited mismatch with the reference sequence. The number of mismatches can be modified by adjusting the parameters of the alignment software keeping in consideration that each modification of the parameters could result in a modification of the mapping results and, consequently, on the specificity/sensitivity of the alignment.

Most common aligners offer the opportunity to choose between two different methods of analysis and reporting: multiple alignment or best alignment. In the multiple alignment method, all the possible hits in a viral sequence database for a single read are reported by alignment score in descending order. While this method provides the possibility to identify all the possible closely-related species in a sample, it is slow for very large datasets and databases. In the best alignment method, only the best alignment between the read and the reference sequence database is reported. If the read has an alignment score identical for two or more hits, the software automatically decides the reference sequence to report. In this method, the read is only used once, and aligned only on a single reference sequence.

As viruses diverge, the analysis of short reads may no longer be sufficient to identify weak sequence homology. Assembling overlapping reads into longer contiguous sequences (contigs) may help identify highly divergent viral sequences, since weak homology extending over the longer contigs results in significant alignments, in contrast to shorter single reads. De novo assembly may, therefore, be needed to detect divergent viral sequences (Figure 2A). De novo read assembly can require significant computational resources, especially if a counter-screen was not employed to reduce the starting number of reads (see section Reference subtraction and counter-screen). The quality of the viral sequence assembly depends on a number of factors, including the amount of virus (which ultimately affects the coverage of the genome); the genome size and type (linear or circular); library preparation method used; the sequencing platform; number of reads; and assembly program(s) [48]. Due to this complexity, and the different quality metrics that can be employed, there is currently no one-size-fits-all optimal sequencing and assembly strategy. Programs used for de novo assembly include ALLPATHS-LG [49], SOAPdenovo [50], and ABySS [51], that appear to be among the most accurate assembly programs for multicellular eukaryotes [48]. Other assemblers, such as SPAdes [52], Velvet [53], or assembler pipelines such as RAMPART [54], A5 [55], or Metassembler [56], are also popular.

The challenges inherent in genome assembly can be offset by the resulting reduction in data volume. Assembly can reduce data volume to as little as a few percent of its original size, depending on coverage. This, in turn, considerably reduces the computation required for mapping sequences to the target database, and increases the likelihood of a significant match. Singlets (single reads) and/or contigs can be searched against viral databases, using tools such as BLAST [19] or BLAT [20]. BLAST is more robust than read-mapping tools in this context, because it is designed to handle sequence divergence, namely, between the sequences in the database and those of actual viruses. This capability can be modulated through different parameters (e.g., distance matrices). Furthermore, BLASTX and/or TBLASTN, which consider translations of the nucleotide sequences, may offer additional sensitivity, owing to the greater conservation of protein sequences, relative to nucleotide sequences (Figure 2B). In each case, query sequences can be masked for repeats using, for example, BLAST’s built-in DustMasker tool [57], or others, such as RepeatMasker [58]. Reads, which failed to assemble, can be mapped against a viral nucleotide database using tools such as Bowtie2, BWA, or SOAP2. However, as mentioned, these software tools typically tolerate only limited sequence divergence and may miss more distantly related viral hits. Reads may also be mapped against a viral protein database with, e.g., RAPSearch2 [59] or DIAMOND [21], which is somewhat more robust, since it operates at the translated sequence level.

### 4.2. Database Selection

The completeness of the database with regard to the virome and accurate annotation of sequences is critical for virus detection. The database enables the assignment of reads based upon sequence identity to viruses, as well as to other organisms, although unmapped reads representing novel organisms not represented in current databases may remain. As mentioned above, in addition to nucleotide sequence analysis, it is also important to query databases based on protein sequences for detection of sequences distantly-related to known viruses (Figure 2B). Protein sequences are more conserved than nucleotide sequences and, thus, might reveal novel organisms that have diverged considerably from existing references. There are a considerable number of options in selecting the database(s). Choices of databases likely depend on whether one wishes to classify only viral or also other microbial sequences, such as bacteria, fungi, or protozoa.

There is a variety of public databases available for the analysis of HTS read datasets, but the most comprehensive of these is the non-redundant nucleotide database “nr/nt” hosted by the National Center for Biotechnology Information (NCBI) [60]. This database includes mostly archival nucleotide data submitted to GenBank and other databases that comprise the International Nucleotide Sequence Database Collaboration (INSDC) [61,62]. However, in addition to viral sequences, nr/nt contains other microbial sequences, as well as cellular sequences, which contribute to the abundance of cellular sequences in this database. The UniProt consortium hosts a protein database derived from INSDC data that has both an archival component (UniProtKB/TrEMBL) and a manually curated component (UniProtKB/Swiss-Prot) [63,64]. This second, curated component, is also included in the NCBI “nr” non-redundant protein database, along with CDS (CoDing Sequence) translations from GenBank data and sequences from other curated sources [60]. Although the data available from nt and nr are comprehensive, these datasets include archival data that have not undergone extensive validation, and may lead to incomplete or misleading findings. High quality datasets, like UniProtKB/Swiss-Prot, have the potential to provide more accurate information about query sequences. Furthermore, extensive, well-curated nucleotide and protein reference datasets are available as part of the NCBI RefSeq project [65]. The virus component of the RefSeq project maintains one or more annotated reference genomes for each viral species and provides a second, reference “neighbor” dataset, which includes representative viral genomes that are validated, but may not be annotated [66]. Although these datasets are updated in real time as new sequences are available, and are generally released every two months, the viral RefSeq data model is generally restricted to full-length genomes present in INSDC databases. Hence, these datasets do not include sequence diversity that may be present in subgenomic length viral fragments or endogenous (integrated into host genome) viral sequences, which are not represented by discrete sequence records.

During routine operation, analysis pipelines access databases that are “housed” locally, rather than accessing them directly from the public resources. These local, custom databases are constructed from the various resources to best fit the intended applications by selectively downloading, applying filter criteria, and/or applying additional annotations. These criteria may limit the database to viruses and virus-like elements, or may address the broader biological world, as mentioned above, to enable the pipeline to account for as many reads as possible. Other choices include whether or not to include partial sequences, which could increase diversity of coverage, sequences with some arbitrarily high degree of similarity, or viral sequences from metagenomics surveys that are accepted by the International Committee on Taxonomy of Viruses (ICTV) [67].

Uncurated sequences in databases are the source of several common problems. Non-viral vector or animal or other host sequences may be present in addition to the viral sequence, resulting in hits that must be considered individually to assess specificity. For example, endogenous viruses, such as retroviruses, are integrated into host cell DNA and, therefore, contain flanking host nucleic acids in addition to virus nucleic acids. Taxonomic information may be lacking, inaccurate, or inconsistent, leading to difficulty in correctly using the information. This is especially a concern for retrovirus elements that may be of interest for possible novel virus detection, but are not immediately obvious if they lack “viral” annotation. For instance, the majority of endogenous retroviruses are currently annotated with their host taxonomy, rather than source virus taxonomic information. However, some endogenous retroviruses are annotated as family *Retroviridae*. It can be difficult to differentiate between endogenous and exogenous retroviruses, since even some viruses annotated with host taxonomy may have both exogenous and endogenous forms. In the absence of formal taxonomy, expert knowledge of virus names/acronyms is often needed to fully understand the characteristics of a particular virus. One approach to development of a comprehensive viral database has been used by Goodacre et al. [68], based on semantic selection criteria to include all viral, viral-like, and viral-related sequences, regardless of length and species, excluding bacterial viruses, and with an overall reduced cellular content. The nucleotidic reference viral database (RVDB) has also been converted to proteic databases, to provide greater flexibility for selection of the best suited pipeline for virus detection. Both are routinely updated and available for public use [69].

### 4.3. Reference Subtraction and Counter-Screen

Some analysis pipelines might use reference subtraction (host genome read removal) and/or counter-screening to reduce the number of “false positive” virus hits, by removing reads that can be better attributed to non-viral sources (Figure 2C). Reference subtraction describes the mapping of sequence reads to a host genome or set of genomes that are known (or assumed) to lack sequences of interest (adventitious viral sequences, in this case). The “non-reference-mapped” reads are then used to query a viral database. Importantly, reference subtraction can be an effective means of significantly reducing the size of a sequence read dataset if it is likely there is residual host cell nucleic acid in the sample, so that the alignment against a viral database would run faster, due to reduced volume. Counter-screening describes either a concurrent alignment of reads against a viral and reference or other broad databases, and retaining the best matches, or alignment of reads first against a viral database, and then the alignment of this smaller set of putative viral reads against non-viral genomes, such as host and bacterial genomes.

Since there may be viral sequences of interest within the reference genome(s) used for subtraction, such as retroviral proviruses or endogenous retroviruses (ERVs) and endogenous viral elements (EVEs), reference subtraction or counter-screening may remove such signals from consideration, creating a blindspot in the analysis. Experimental design plays an important role in deciding whether reference subtraction or counter-screening against the reference genome should be included in an analysis pipeline. If there is an interest for endogenous retroviruses and other endogenous viruses, then their signal within the sequence dataset should be retained by not including reference subtraction for further evaluation. A reference subtraction is still possible in that case, if viral sequences within the reference genome are first masked bioinformatically. This permits non-viral sequences to be subtracted from the dataset specifically, without the risk of hiding viral signals. Subsequent counter-screening with the unmasked reference genome can then determine, if desired, which viral signals identify as both host and virus, indicating an endogenous source.

Counter-screens might well include genomes beyond that of the host cell and, in fact, may include all of the nt or nr databases. The broadest of these counter-screen alignments can be extremely computer-intensive and, usually, must be done with significantly reduced datasets, unless computing infrastructure and/or time are not limiting factors.

Most of the concerns expressed above about creating potential blindspots in the analysis algorithm through reference subtractions and counter-screens can be addressed, as long as the reads that are screened out are somehow further processed to reveal potential novel viruses. We are increasingly aware of the need to further study unmapped reads (see section Unmapped Sequences), but we should also remain aware of the possibilities of blindspots resulting from subtractions, particularly for retroviruses and other endogenous virus sequences. A well-characterized reference genome may reduce or eliminate this concern. Also, ideally, we would attribute all reads to their top best matches; this remains a computational and algorithmic challenge for many.

### 4.4. Processing of Viral Hits

Once a pipeline has produced a list of hits, these must be understood individually, in order to prioritize and facilitate downstream follow-up to verify and further investigate the results.

First, the analyst must make a decision as to which hits are actionable. If host subtraction and counter-screening are designed appropriately in upstream steps, the set of hits at this stage of the analysis should contain only taxa for which there is reasonable evidence for their presence. In some cases, however, the list will include multiple conflicting taxa derived from the same set of reads. If no strong evidence exists in favor of one or the other alternative hypothesis, further analysis should be considered.

Additionally, the analyst may evaluate the putative hits for “expected” false positives. Experience has shown that there are a few matrix-specific false positives, which recur in these analyses, and these can be flagged as low-risk (although the team may still decide that they warrant follow-up). Such known hits include signals coming from reagents, kits, misannotated sequences, and low-complexity sequences. In the future, it would be useful to initiate a collaborative effort across organizations to pool and evaluate data from viral screening experiments. This could lead to the construction of a database of common false positives and the contexts in which they are likely to appear. The problem of reagents introducing viral-like sequences during HTS library preparation can also be handled by confirming their presence by testing the original biological sample by PCR assays using alternative reagents unlikely to contain the same contaminant. Additionally, rigorous procedures should be used at all steps to avoid cross-contamination from other samples processed in the same facility.

There is reason to consider the length or the percentage of total genome covered in addition to the number of reads or contigs matching a viral sequence. For instance, a common spurious result is a narrow but very deep coverage that could be due to a single conserved motif, engineered vector elements shared by viruses, such as a polyomavirus SV40 promoter, or to an artifact of sequencing. These results can be somewhat managed through evaluation of the quality and length of coverage. For example, hits that meet a minimum coverage threshold of perhaps 100 or 200 nucleotides, with 80% or 90% identity, may detect known viral sequences. This dramatically reduces the number of hits to evaluate, and experience has shown that the vast majority of these very short coverage hits are non-specific. The most compelling “hits” are arguably those with the longest length of genome coverage, not necessarily the most reads matching what may be a very narrow (and often artefactual) region. Figures showing read coverage and sequence similarity to the best annotated reference genome should be generated.

Once the above steps are complete, the analyst may have a list of viral hits for which no better hypothesis exists. Some of these viral hits may have only one or a few reads supporting them, and the decision could be made that this evidence is insufficient to warrant follow-up depending on the stringency of the match. However, dismissing weak signals of this type may be unadvisable with additional follow-up. Furthermore, a strong sequence match, even based on a single read, is unlikely to happen by chance.

Follow-up may include trying to extend the sequence(s) matching a virus. To do this, one can perform a reference-guided assembly on the starting read set, using the most closely matched genome already identified. This approach can generate a longer, more correct sequence, which can then serve as a template for further alignment, as well as for the design of downstream assays. Matches to different region of the same virus (or viral family) in the same sample can also be connected by PCR assays to generate a longer region of the viral genome. This type of approach was used to identify a novel rhabdovirus that was unexpectedly present in an insect cell line [5].

Additionally, at this stage, the analyst can gather more information to determine the biological significance of the hits. This would include expected tropism, novelty relative to database species, diversity of the viral family, and pathogenicity.

Exact rules or conditions to consider a bioinformatics hit as a true positive is currently an area of active discussions, and is yet to be determined. If contamination during sample preparation can be excluded, and viral presence confirmed by PCR, a single read with a nearly perfect match to a known virus could be considered strong evidence of its presence in the tested sample. Whether the virus is of actual concern would depend on further follow-up to assess potential risk and discussion with regional regulatory authorities.

### 4.5. Unmapped Sequences

A metagenomics pipeline for the identification of viruses and/or microorganisms mainly depends on mapping sequences to complete or partial genome sequences. The previous sections described the possible methods to apply for sequences having nucleotide sequence hits in a database. However, it is often reported in such analyses that several reads (or assembled contigs) do not map to any known nucleotide sequences. These unmapped sequences require a separate bioinformatics pipeline to determine their origins (Figure 2D). Some cannot be mapped for trivial reasons, for example, low-complexity sequences whose match score e-value may remain above threshold, despite high similarity to a reference. Those can be caught upfront using bioinformatics filters, and need not to be categorized as risks which require follow-up. Some unmapped sequences can belong to the host but not be included in its genome assembly, such as centromeric sequences, which are difficult to assemble [70]. Some unmapped reads may originate in species whose genomes are not yet available, including novel viruses. Although sequence databases continue to grow exponentially, it is clear that there remain novel sequences yet to be discovered.

For pipelines based on read mapping, the unmapped reads need to be assembled into contigs, or otherwise clustered (for sequences that are difficult to assemble), to detect whether the reads have single or multiple origins. A robust contig in one sample should be detectable in another sample by alignment if it is similarly present. Therefore, a negative control sample can be used to identify contigs that are unique to the tested sample. These contigs can be mapped to possible species level sequences by relaxing the parameters for sequence matching. Nucleotide k-mer-based approaches can assign species based on the presence and frequency of specific k-mers (short nucleotide substrings).

The frequency of a set of k-mers in a species’ genome, in a genomic region or in a class of sequences, can be used as a “signature” of the underlying sequence. Comparing these frequencies is computationally easier than sequence alignment, and is an important method in alignment-free sequence analysis. The k-mer-based method is implemented in tools such as CLARK [71] or Kraken [31], GC-content is used in TAC-ELM [72], and oligonucleotide frequencies are used in TACOA [73], MetaID [74], or AKE [75]. When building classifiers, these features could eventually be extended by estimated open reading frame (ORF) length or/and density, codon usage, motifs, or repeats, such as microsatellites, transposons, or CRISPRs (clustered regularly interspaced short palindromic repeats) that could help to differentiate viral from non-viral sequences.

The length of sequences is critical to the classification accuracy of these latter techniques; therefore, they could be used only on contigs. Meanwhile, k-mer frequencies used in LikelyBin [76] and AbundanceBin [77] achieved good accuracies on short reads.

The presence of conserved functional domains at the protein level can also give hints about the taxonomic assignment. Databases such as Pfam [78], CDD [79], SMART [80], KOG [81], COG [82], Protein Clusters [83], or TIGRFAMs [84] can be screened using specific tools to find hints about the conserved functions potentially present in assembled contigs.

As described in the section below “Data Captured in Raw Output”, a complete visualization of the species level assignment should be given using a phylogenetic tree diagram to communicate the degree of taxonomic resolution obtained.

If a sequence defies taxonomic identification, it can be retained for reanalysis once reference databases are updated or expanded, and can also be added to an “orphan” database against which subsequent datasets can be searched. If seen again, the sequence contigs can be grown, perhaps to the point of being identifiable or otherwise understood. Novel viruses may have sequence segments that have diverged, preventing taxonomic identification, but some segments may be identifiable or able to be positioned in the known sequence space for viruses. Using assembly and paired-end read information, unmapped sequences may become linked to sequences of known origin.

Finally, risk can be further mitigated by running a suitable negative control alongside the analyzed samples. Should the unidentified sequences also be present in the negative control, then the analyzed samples would not likely be the source of this signal. Managing the collection of unidentifiable sequences can, therefore, take advantage of information about the composition of analyzed samples, in addition to any information collected about the sequences during their characterization. By definition, the information would be too weak, individually, to support identification, but collectively (perhaps by using machine learning approaches), may provide plausible leads that could be further explored.

## 5. Data Captured in Raw Output

Output from a sequence identification pipeline not only depends upon the databases against which sequences are searched, but also upon the strategies used for filtering the raw data, for searching the databases, and for counter-screening the primary hits. In any case, the sequence identification pipeline will produce a listing of sequences that match one or more taxonomies above some threshold measure of confidence, and a listing of sequences that do no match any taxonomies above that threshold.

Ideally, each sequence will be grouped together with all other sequences matching a given taxonomy, in order to produce a read count. These sequences will match their respective viral references with different degrees of confidence, from just above the threshold of acceptance, through to identity with the reference. Statistics derived from this distribution, such as mean and standard deviation, can contribute both to the confidence of the collective assignment of the set of reads, as well as to an assessment of how different the putative contaminant might be from the reference sequence. The latter is especially important in follow-up risk assessments, guiding predictions about the impact of the contaminant in manufacturing, and product safety.

Different strategies can be envisioned for setting thresholds above which a match should be listed, and will depend upon the strategy used in sequence identification. Obvious candidates for measuring confidence include some minimum percentage of identity and/or coverage between the read and reference sequences, tool-specific match scores (e.g., bit score, e-value, *p*-value, or alignment score), or minimum read counts. Strategies beyond these primary measures can also be employed in order to improve upon the signal-to-noise ratio, such as competitive matching (e.g., when a counter-screen strategy is employed) or any number of statistical treatments of the primary match score distribution.

Often, assignments to a given taxonomy may be ambiguous, where the match, within some set tolerance, might be to any number of related or unrelated references. When the match profiles to related references cannot be resolved, the match can simply be escalated to the higher taxonomic group to which those references belong. Displaying such assignments on a phylogenetic tree may be helpful to communicate the degree of taxonomic resolution obtained. Alternatively, in a method such as TAMER [85], sequence reads are assigned to the candidate genomes, and the taxonomy tree based on the estimated probability, by taking into account both sequence alignment scores and estimated genome abundance.

On the other hand, when equivalent match profiles against unrelated references are observed, the alternative assignments should all be reported, appropriately weighted based on the evidence, to the risk analysis team. Conflicting assignments may occur due to chance or due to convergent evolution (typically, when match scores are very low or when sequences have low complexity), or due to lateral gene transfer, where genetic material has moved between species. The most relevant of the gene transfer events that pipelines might discover concern the integration of viral or microbial sequences within a host cell. In such cases, a sequence is, for example, both viral and host, and pipelines should not report just one or the other, since risks differ between host (no risk), integrated virus (some risk), and exogenous virus (more risk).

A confident set of matches to a reference sequence might still not indicate contamination: the sequences may be from just one specific segment of the reference sequence, such as a human cytomegalovirus (CMV) promoter engineered into a cell line, an oncogene present in the host and shared with a retrovirus, or any other such lateral gene transfer event. Mapping sequences to the virus reference is therefore indicated, in order to understand the nature of the signal, and more practically, in order to derive a consensus sequence for the signal from which further experiments may be launched, for example, to determine if full-length virus is present in the analyzed sample by long-range PCR.

## 6. Final Reporting Format

Different organizations will have different formats for the final report but, in all cases, it is beneficial to establish a consistent and standardized reporting format, which clearly communicates the analytical findings, as well as the strength of the evidence and the quality of the underlying data. It is also beneficial if most of the report’s content can be automatically generated.

We suggest that the top-level findings include the following: (1) whether or not contaminants were found, (2) identity of contaminants, (3) “strength” of the evidence, and (4) algorithm adopted for the alignment of the reads. Strength of evidence includes alignment score, depth and sequence coverage, e-value, etc. This information should be communicated in an executive summary at the beginning of the report.

In addition to the executive summary, the report should contain one section for each identified contaminant, including the number of reads supporting its identification, a figure showing the coverage and sequence similarity to the nearest reference genome, and a phylogenetic tree or other view demonstrating related hit taxa in the event that an unambiguous call cannot be made. Comments from experts on the subject, together with the likelihood of the findings, could be included, if appropriate, to the organization’s decision-making flow. It would be suitable to generate dynamic and interactive reports, where the front-end users could select reported hits and summarize the findings in table/figure formats.

Information about the overall experiment, including technical and bioinformatics details, should also be captured. This would include the list of samples appropriately linked to their provenance, experimental details, including the nucleic acid and library preparation methods, and information about the sequence data, such as the total number of reads, the sequence quality, and the number of reads that aligned to host sequence when the host sequence is known and utilized in the analysis. Additionally, details regarding the bioinformatics tools and parameters used for the analysis, including databases along with version numbers, should be documented. When possible, a link to the raw and processed read data should be provided. The final report should summarize the potentially complex experiment such that the audience can easily grasp the findings and their potential significance. Since we are in the early stages of HTS applications, discussions should continue for developing “best” practices for data submission.

## 7. Conclusions

The experimental and computational issues related to adventitious agent detection using HTS have been discussed, including experimental design (to a limited extent), factors related to detection sensitivity, sequencing platform and file format, data processing, database and reference genomes, de novo assembly and read mapping algorithms, post-processing of unmapped reads, and final report format.

Using HTS to identify adventitious viruses offers advantages over other biological methods or assays, by allowing quick and broad detection of virus in the absence of known sequence information for study design. To evaluate a sample for adventitious virus by HTS, the experimental design should take into consideration various factors that influence the detection sensitivity and specificity, i.e., total read yield, potential virus type, library prep and bioinformatics pipeline, etc. Most commercially available HTS platforms exhibit effectiveness to detect adventitious virus in biological samples, while platforms yielding higher number of sequence reads have a greater likelihood of detecting low level virus contaminants. The pre-processing and alignment suites offered by commercial HTS providers, such as Illumina and Ion Torrent, have been found to be useful. FASTQ file is used as the most frequent input file format by many sequencing mapping algorithms, such as Bowtie2, BWA, and is considered the standard file format. When de novo assembly is applied to the pre-processing step, the assembled sequence contigs in FASTA file format is then used in the downstream pipeline analysis. In addition to de novo assembly, other sequence pre-processing approaches, such as removing host genome reads, can help to reduce the input sequence file size and, thus, improve the downstream computational performance. However, in most situations, whether to remove reads related to the host genome may also depend on the analysis preferences or objectives.

The reference database plays an important role in the bioinformatics pipeline for adventitious virus detection, therefore, it is critical to curate and construct non-redundant, high quality, and accurately annotated virus databases [68]. In addition, the detection sensitivity can also be related to the bioinformatics workflow, not only the mapping algorithms but also the settings of corresponding parameters of each algorithm. Further post-alignment of unmapped reads using different methods (i.e., sequence assembly, machine learning, etc.), followed by biological interpretation, will also aid in the detection of new adventitious virus, and confirm the presence of viruses. Finally, the study report should include the biological interpretation, as well as information such as total number of sequence hits, the coverage and depth of the viral genome, and annotated information, such as taxonomy classification.

The major strategies and common shared points discussed here are all based on the current stage of technology development and discovery. As the sequencing platforms and software tools continue to advance, it can be surmised that the methods and bioinformatics pipelines will also continue to improve, yielding enhanced assays for the detection of low-level contaminating adventitious agents.
